# Clinical characteristics, management, and outcomes of cadmium poisoning: a systematic review of case reports and case series

**DOI:** 10.3389/fpubh.2025.1651851

**Published:** 2025-09-10

**Authors:** Zekai Shao, Minyan Wang, Oscar Onayi Mandizadza, Conghua Ji

**Affiliations:** School of Public Health, Zhejiang Chinese Medical University, Hangzhou, China

**Keywords:** cadmium poisoning, case report, chelation therapy, blood cadmium, urine cadmium

## Abstract

**Objective:**

This paper synthesizes case reports and series to outline the clinical features, diagnosis, treatment, and prognosis of cadmium poisoning. It offers structured evidence to improve understanding, early diagnosis, and clinical management, aiding healthcare professionals in prevention and intervention.

**Methods:**

The study protocol has been registered in the PROSOPERO system (CRD420251128280). We independently searched PubMed, Scopus, and Cochrane databases using keywords including “cadmium poisoning,” “case report,” and “case series.” Only English-language case reports and series with confirmed or highly suspected cadmium poisoning were included, up to May 2025. Animal studies, reviews, and articles lacking individual patient data were excluded. Additional references were identified from the bibliographies of retrieved articles. Study quality was assessed using the JBI tool for case reports/series.

**Results:**

Among the 12 included studies, there were a total of 17 patients. We found that cadmium poisoning primarily affects adult males and is often associated with occupational exposure. Common risk factors include smoking and alcohol consumption. The study also found that low iron stores exacerbate cadmium poisoning. Diagnosis relies on a combination of clinical diagnostic tests, blood and urine tests, chest X-rays, kidney ultrasounds, bone density measurements, and skeletal imaging. The treatment plan includes the use of chelating agents to reduce cadmium levels in the body and antibiotics to maintain the patient’s condition. For patients with concurrent lung and kidney involvement, mechanical ventilation and continuous renal replacement therapy (CRRT) may be required. Additionally, for patients with osteochondropathy, supplementation with calcium and vitamin D is recommended. While most patients have a favorable prognosis, fatal cases due to respiratory failure or renal failure have been reported.

**Conclusion:**

This review emphasizes that cadmium poisoning is rare and complex, with non- specific symptoms and a tendency to cause organ damage. Therefore, it is necessary to enhance clinical vigilance. Occupational exposure highlights the need for public health surveillance. Diagnosis relies on blood or urine cadmium testing, which is crucial for guiding treatment. Although the condition is relatively uncommon and most patients have a favorable prognosis, the risk of severe complications and mortality underscores the importance of timely and individualized treatment. Further research is needed to optimize management and improve long-term outcomes.

**Systematic review registration:**

(registration number: CRD420251128280).

## Introduction

Exposure to cadmium (Cd poisoning) is relatively rare but potentially life-threatening. The incidence is significantly higher in populations exposed to welding or industrial work involving cadmium, making it a growing concern for both public health and occupational safety. Although rare, the risk of developing complications and death increases significantly with inappropriate or delayed treatment. For instance, a study of 7,131 cadmium workers, conducted in the United Kingdom, reported 1,105 deaths (1). In a study of biochemical analysis in a population with high cadmium exposure, 5 out of 27 copper smiths exposed to cadmium fumes (18.5%) had kidney stones. Compared to the control, they showed evidence of kidney damage through blood biochemistry and proteinuria (2). In a study of amino aciduria among Japanese workers in the cadmium industry, amino aciduria was elevated to varying degrees, with differing impacts on kidney function (3). Research indicates that cadmium not only harms occupational populations but also has a significant impact on local populations in areas with high cadmium concentrations. A study in the Yatağán coal mining region of Turkey demonstrated that cadmium also affects metal levels in local children (4). Early detection and management are essential to prevent adverse outcomes. Studies have explored several mechanisms that increase the risk of cadmium accumulation in the body. For instance, smoking can potentially increase cadmium absorption in the lungs, indirectly leading to increased cadmium load in the body (5). Alcohol intake can also increase the gastrointestinal absorption of cadmium (6). In addition, iron deficiency is a risk factor for increased blood and urine cadmium (7).

Clinically, cadmium poisoning can manifest a variety of symptoms, some of which are atypical, often leading to delays in diagnosis and treatment, which may result in worsening of the condition. Common symptoms include cough, respiratory distress, and fever. In addition, it also presents with diarrhea, joint pain, and elevated urea and creatinine levels (8, 9).

The diagnosis of cadmium poisoning relies heavily on the physician’s clinical assessment and routine biochemical tests. However, the atypical symptoms of cadmium poisoning also increase the difficulty of diagnosis. Therefore, biochemical examinations are particularly important. Measuring cadmium concentration in blood and urine helps determine the severity of cadmium poisoning.

Cadmium poisoning is primarily treated using chelating agents that help to remove cadmium ions from the body; however, the safety and effectiveness of this treatment option remain controversial (9, 10). Since cadmium poisoning has a similar clinical presentation to pneumonia, doctors might initially administer treatment for pneumonia if cadmium exposure is not known, missing a critical treatment window, leading to a poorer prognosis (11).

This systematic review integrates evidence from published case reports and case series studies of patients diagnosed with cadmium poisoning to comprehensively describe their clinical characteristics and diagnostic strategies, summarize management approaches including chelation therapy and supportive care, and report on patient outcomes, including recovery, mortality, and sequelae. This review provides a structured overview, offering clinicians and public health professionals a structured summary of evidence to enhance awareness of cadmium poisoning, promote early diagnosis and timely intervention, and provide guidance for clinical management and preventive measures.

## Methods

### Protocol and registration

This systematic review follows the reporting guidelines for systematic reviews and meta-analyses and involves a secondary analysis of published studies (12). The study protocol has been registered in the PROSOPERO system (registration number: CRD420251128280).

### Literature search

We developed our search strategy in consultation with a subject specialist to ensure completeness and relevance. The strategy used a combination of Medical Subject Headings (MeSH) terms and free-text keywords, incorporating terms such as “cadmium poisoning,” “case report,” and “case series.” The databases searched were PubMed, Scopus, and Cochrane, covering publications up to May 2025. The search was limited to articles published in English. Reference lists of relevant articles and reviews were also screened to identify additional studies.

### Research quality assessment

We used the JBI checklist to assess the quality of the included case reports. The JBI checklist was originally described by Munn et al., and it provides a structured framework for assessing the risk of bias and reporting adequacy in case-based evidence.^1^ By applying this tool, we ensured that the evidence included in this review was critically appraised in a standardized and transparent manner.

### Study selection

The retrieved studies were imported into EndNote 20.0 (Clarivate Analytics, Philadelphia, United States) for duplicate removal and screening. Two independent researchers screened study titles and abstracts, followed by a full-text review of potentially eligible studies based on the inclusion and exclusion criteria. Any discrepancies were resolved through discussion, with adjudication by a third researcher when necessary.

### Inclusion and exclusion criteria

The inclusion criteria for this overview were as follows: (1) the study population consisted of patients with cadmium poisoning; (2) the study was either a case report or a case series.

Articles were excluded if: (1) The study population was not the target population, (2) non-target interventions were performed on the target population. (3) Methodological flaws in the study (inadequate diagnosis, abnormal termination of treatment follow-up), or (4) Missing data: Data on core variables (such as exposure levels and primary outcome measures) are missing from the study and cannot be obtained by contacting the authors. (5) Contradictory results: The results of clinical diagnosis and treatment contradict the theoretical expected results.

### Data extraction

Data extracted from each study included:

Study characteristics: author(s), year of publication, and country of study.

- Patient demographics: age, gender, adverse lifestyle habits and occupation.
- Clinical manifestations: signs and symptoms at the time of consultation.
- Diagnostic methods: clinical diagnostic tests and biochemical tests, etc.
- Treatment modalities: conventional treatment of cadmium poisoning, pulmonary ventilation, renal replacement therapy (CRRT).
- Outcomes: clinical outcomes (recovery and relapse), complications, sequelae, mortality, and duration of follow-up.

Two independent reviewers extracted data using a standardized form. Discrepancies were resolved through discussion or consultation with a third reviewer.

### Statistical analysis

We used Microsoft Excel (Microsoft Corp, Seattle, WA, United States) for data collection and organization. Furthermore, we used descriptive statistics to summarize the raw data, presenting the means and standard deviations for continuous variables, while frequencies and percentages were reported for categorical variables.

## Results

### Literature search

**Figure 1:**
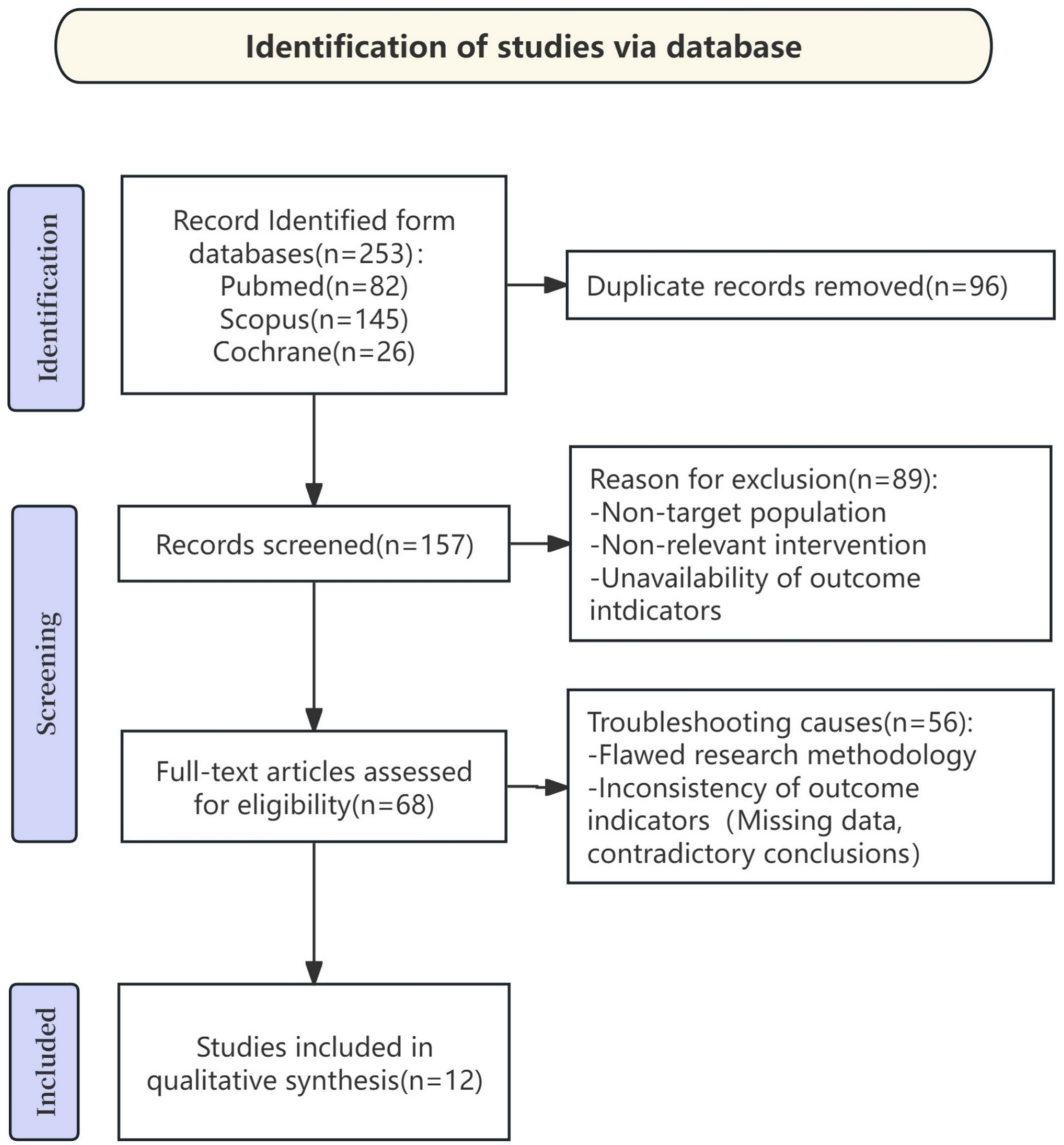
Flowchart of literature screening.

A total of 253 documents were retrieved using the specified search terms “cadmium poisoning,” “case series,” and “case report.” After excluding 96 duplicate documents, 157 documents were included in the full-text review. Among these, 145documents were further excluded due to reasons such as the study subjects not being the target population, inconsistent outcome measures, and missing data. Ultimately, 12 documents met the inclusion criteria and were included in the systematic review. The detailed screening process is shown in Figure 1.

### Research quality assessment

We conducted a comprehensive assessment of the 12 included documents. After independent evaluation, all 12 studies met the inclusion criteria, and their methodological quality was considered reliable. The detailed appraisal outcomes are presented in Table 1. It should be noted that this review is based solely on 12 case reports/case series, with a limited sample size, which to some extent limits the representativeness and generalizability of the results.

**Table 1:** Conduct a JBI checklist review of the case reports.

Literature	Patient	JBI checklist								
		1	2	3	4	5	6	7	8	Include
R H Townshend/1982 (15)	Patient 1	Y	Y	Y	Y	N	Y	Y	Y	Y
Richard Wittman/2002 (18)	Patient 2	Y	Y	Y	Y	N	Y	Y	Y	Y
Yuichi Ando/1996 (11)	Patient 3	Y	Y	Y	Y	Y	Y	Y	Y	Y
J D Blainey/1980 (34)	Patient 4	Y	Y	Y	Y	Y	Y	Y	Y	Y
Ayan Roy/2024 (9)	Patient 5	Y	Y	Y	Y	Y	Y	Y	Y	Y
Kristin Seidal/1993 (14)	Patient 6	Y	Y	Y	Y	Y	Y	Y	Y	Y
R. H. Townshend/1968 (13)	Patient 7	Y	Y	Y	Y	Y	Y	Y	Y	Y
Hector P. Blejer/1966 (16)	Patient 8	Y	Y	Y	Y	Y	Y	Y	Y	Y
	Patient 9	Y	Y	Y	Y	Y	Y	Y	Y	Y
D. C. Beton/1966 (23)	Patient 10	Y	Y	Y	Y	Y	Y	Y	Y	Y
	Patient 11	Y	Y	Y	Y	Y	Y	Y	Y	Y
	Patient 12	Y	Y	Y	Y	Y	Y	Y	Y	Y
	Patient 13	Y	Y	Y	Y	Y	Y	Y	Y	Y
	Patient 14	Y	Y	Y	Y	Y	Y	Y	Y	Y
A. Taylor/1984 (8)	Patient 15	Y	Y	Y	Y	Y	Y	Y	Y	Y
D H Yates/1990 (5)	Patient 16	Y	Y	Y	Y	Y	Y	Y	Y	Y
ZHOU Yuan-shen/2022 (17)	Patient 17	Y	Y	Y	Y	Y	Y	Y	Y	Y

1. Were patient’s demographic characteristics clearly described? 2. Was the patient’s history clearly described and presented as a timeline? 3. Was the current clinical condition of the patient on presentation clearly described? 4. Were diagnostic tests or assessment methods and the results clearly described? 5. Was the intervention(s) or treatment procedure(s) clearly described? 6. Was the post-intervention clinical condition clearly described? 7. Were adverse events (harms) or unanticipated events identified and described? 8. Does the case report provide takeaway lessons?

### Demographic characteristics

The 12 included articles reported a total of 17 cases of cadmium poisoning. Among these cases, patients ranged from 23 to 78 years of age. Most cases occurred in the 30 to 50 years age group and were predominantly male, which aligns with the typical age and gender distribution of welders and metalworkers. Occupational exposure is the primary cause of cadmium poisoning. The vast majority of patients with cadmium poisoning usually have an occupational history of exposure to cadmium. However, poor lifestyle habits are important factors that contribute to poor prognosis in cadmium poisoning. Prolonged smoking can increase urinary cadmium concentrations and the risk of long-term complications. Men who smoke and drink alcohol are also at higher risk of developing complications. In addition, low iron stores in the body enhance cadmium absorption. Case report information and patient demographic characteristics are shown in Table 2.

**Table 2:** The demographic characteristics of the cases.

literature	Patient	Age	Sex	Country	Profession	Disease	Bad habit	Past medical history
R H Townshend/1982 (15)	Patient 1	51	Male	UK	Welder	Acute cadmium pneumonia	Smoking (3–4 cigarettes/day)	No specific medical history
Richard Wittman/2002 (18)	Patient 2	28	Female	USA	Jewelry Manufacturing Processing	renal tubular damage	Non-smoking	No specific medical history
Yuichi Ando/1996 (11)	Patient 3	43	Male	Japan	Welder	Cadmium pneumonia	Smoking (20 years, 2 packs/day)	10 years ago Automobile factory welder for 1 year (wearing a protective mask)
J D Blainey/1980 (34)	Patient 4	71	Male	UK	Battery plate manufacturing	Chronic cadmium toxic osteomalacia	Low calcium/vitamin D diet, long night shifts	25 years of proteinuria (renal tubular dysfunction), 12 years of bone disease (fractures, deformities), and obesity
Ayan Roy/2024 (9)	Patient 5	40	Male	India	silversmith	Chronic cadmium toxic osteomalacia	Not mentioned	No specific medical history, 5 years of bone pain/muscle weakness (misdiagnosed)
Kristin Seidal/1993 (14)	Patient 6	78	Male	Sweden	toolmaker	Cadmium pneumonia	Non-smoking	Mild angina (long-term sotalol)
R. H. Townshend/1968 (13)	Patient 7	51	Male	UK	Welder	Acute cadmium pneumonia	Heavy smoking	No specific medical history
Hector P. Blejer/1966 (16)	Patient 8	35	Male	USA	Welder	Acute cadmium pneumonia	Not mentioned	No specific medical history
	Patient 9	28	Male	USA	Welder	Acute cadmium poisoning	Not mentioned	No specific medical history
D. C. Beton/1966 (23)	Patient 10	53	Male	UK	Steel erector	renal cortical necrosis	Not mentioned	History of eczema
	Patient 11	42	Male	UK	Welder	pulmonary edema	Smoking (pipe smoker)	Duodenal ulcer
	Patient 12	38	Male	UK	Steel erector	cadmium pneumonitis	Smoking (30 cigarettes/day)	No specific medical history
	Patient 13	35	Male	UK	Steel erector	pulmonary infection	Not mentioned	History of hernia surgery
	Patient 14	39	Male	UK	Steel erector	cadmium pneumonitis	Not mentioned	Recent testicular infections
A. Taylor/1984 (8)	Patient 15	36	Male	UK	Not mentioned	Cadmium poisoning	Not mentioned	No specific medical history
D H Yates/1990 (5)	Patient 16	49	Male	UK	Welder	cadmium pneumonitis	Former smokers (ex-smoker)	No specific medical history
ZHOU Yuan-shen/20220 (17)	Patient 17	23	Male	China	Not mentioned	Acute severe cadmium poisoning	Not mentioned	No specific medical history

### Diagnosis

The most common diagnostic method for cadmium poisoning is laboratory examination. Although respiratory symptoms are often prominent, clinical diagnosis alone is insufficient, as these symptoms are not easily distinguished from those in pneumonia, which can lead to misdiagnosis. Therefore, laboratory tests play a crucial role. Measuring blood and urine cadmium concentrations provides key evidence for confirming cadmium poisoning. In more severe cases, chest x-ray examination and renal ultrasonography are needed to determine organ abnormalities (7). For cases with nonspecific findings, bone density measurements and bone imaging are also needed to determine bone abnormalities (9, 13). For detailed information, please refer to Table 3.

**Table 3:** The clinical symptoms and diagnostic methods of the cases.

Literature	Patient	Clinical manifestation	Diagnostic methods	Judgment criteria
R H Townshend/1982 (15)	Patient 1	**Systemic symptoms:** chills, shortness of breath after activity **Lung:** chest pain **Other:** pestle finger	**Laboratory tests:** **Imaging tests:** chest imaging **Respiratory function tests.**	**Cadmium concentration in urine and blood**
Richard Wittman/2002 (18)	Patient 2	**Systemic: anxiety** **Lungs:** shortness of breath, chest pain **Kidneys:** left-sided dystocia, polyuria	**Laboratory tests:** blood and urine β₂-microglobulin tests **Imaging tests:**renal ultrasound, chest radiographs **Respiratory function tests**	**Blood cadmium, urinary cadmium, β₂-microglobulin**
Yuichi Ando/1996 (11)	Patient 3	**Systemic:** high fever, chills, nausea **Lungs:** labored breathing, coughing	**Laboratory tests:**blood, urine, arterial blood gas analysis **Imaging tests:** chest X-ray, chest CT	**Urinary cadmium, *β*₂-microglobulin**
J D Blainey/1980 (34)	Patient 4	**Skeletal system:** bone pain, deformities, fractures (spontaneous) **Cardiovascular system:** hypertension, atherosclerosis of the aneurysm	**Laboratory tests:** hepatic cadmium, renal cadmium, urinary cadmium, β₂-microglobulin, lysozyme **Imaging tests: skeletal x-rays** **Renal function tests:** proteinuria, creatinine **Special:** Bone Biopsy	**High concentrations of cadmium in tissues (liver/kidney), proteinuria, bone growth**
Ayan Roy/2024 (9)	Patient 5	**Systemic symptoms:** mild anemia **Skeletal system:** generalized bone pain, pseudofractures **Muscular and nervous system:** muscle weakness, spasticity **Kidney:** increased urine output	**Laboratory tests**: routine blood, endocrine-related, routine urine, β₂-microglobulin **Imaging tests:** skeletal X-ray, renal ultrasound, cervical MRI **Respiratory function tests:** renal function assessment **Special**: Bone Biopsy	**Elevated blood cadmium, urinary cadmium concentration, and FGF-23 levels**
Kristin Seidal/1993 (14)	Patient 6	**Systemic symptoms:** fever (38.3°C) **Pulmonary symptoms:** persistent cough, dyspnea, cyanosis	**Laboratory tests**:blood cadmium, renal cortical cadmium, liver tissue cadmium **Imaging tests:** chest x-ray **Special**: Autopsy	**blood cadmium concentration**
R. H. Townshend/1968 (13)	Patient 7	**Systemic symptoms:** fever, weight loss **Pulmonary symptoms:** dyspnea	**Laboratory tests:** blood cell count, plasma protein electrophoresis **Imaging tests:** chest x-ray tomography scan **Physical examination:** slight cyanosis (at rest), fine wet rales at the base of the right lung, weight loss **Pulmonary function tests** **Special:** bronchoscopy and biopsy	**Pulmonary respiratory symptoms + confirmed high cadmium concentration**
Hector P. Blejer/1966 (16)	Patient 8	**Systemic symptoms:** fever, cyanosis **Pulmonary symptoms:** severe cough, chest pain, shortness of breath	**Imaging tests:** chest x-ray **Special:** Autopsy	**Cadmium in urine, cadmium concentration in lung tissue**
	Patient 9	**Pulmonary symptoms:** cough (mild) **Digestive symptoms:** stomach cramps, nausea, diarrhea, abdominal pain	**Laboratory tests:** blood cadmium, urine cadmium **Imaging tests:** chest x-ray **Physical examination:** blood pressure, lungs	**Blood cadmium and urine cadmium concentrations**
D. C. Beton/1966 (23)	Patient 10	Cough, dyspnea, cyanosis, fever	**Laboratory tests:** routine blood, blood biochemistry, urine tests, sputum culture, blood cadmium, urine cadmium **Imaging tests:** chest X-ray, electrocardiogram **Physical examination:**blood pressure, temperature, cardiopulmonary examination **Pulmonary function tests**	**Cadmium concentration in lung/kidney/liver tissue**
	Patient 11	Chest pain, dyspnea		Pulmonary imaging shadow
	Patient 12	Night sweats, muscle aches, diarrhea		Pulmonary imaging shadow
	Patient 13	Sore throat, knee pain, hematuria		sputum culture
	Patient 14	Chills, chest pain, vomiting		Identify high cadmium concentration exposure
A. Taylor/1984 (8)	Patient 15	**Systemic symptoms:** severe headache, generalized myalgia, mild confusion, dehydration **Lungs:** dyspnea **Digestive**: severe vomiting, watery diarrhea, diffuse abdominal pain **Urinary system:** drastic decrease in urine output	**Laboratory tests**: routine blood, electrolytes, renal function, blood cadmium, urine cadmium **Imaging tests:** chest X-ray, electrocardiogram (ECG) **Special**: autopsy analysis	**Blood cadmium and urine cadmium concentrations**
D H Yates/1990 (5)	Patient 16	**Systemic symptoms:** fever, malaise, arthralgia **Respiratory symptoms:** shortness of breath, cough, hemoptysis	**Laboratory tests:** blood tests, blood sedimentation, autoantibodies, urine tests, blood cadmium, urine cadmium **Imaging tests:**chest X-ray **Physical examination:** wet lung rales **Lung function tests**	**pulmonary shadow**
ZHOU Yuan-shen/20220 (17)	Patient 17	**Systemic symptoms:** sudden loss of consciousness, twitching of limbs, cold sweats, hypotension **Respiratory symptoms:** respiratory failure **Urinary symptoms:** oliguria	**Laboratory tests:** urinary cadmium, blood cadmium **Imaging examinations:** chest X-ray and CT, echocardiography, abdominal CT **Special:** Detection of organ damage markers (serum creatinine, alanine aminotransferase, aspartate aminotransferase, brain natriuretic peptide, prothrombin time, D-dimer)	**Blood cadmium and urine cadmium concentrations**

### Complications in cadmium poisoning

Complications in cadmium poisoning are multifaceted and involve several body organs and tissues. The most common complications involve the lungs and kidneys. Inhalation of high concentrations of cadmium fumes can damage capillaries and lead to acute cadmium pneumonia (14). Chronic low-dose cadmium exposure may result in chronic obstructive pulmonary disease (COPD) with pulmonary fibrosis (15). In the kidneys, cadmium toxicity primarily causes tubular damage by disrupting cellular homeostasis, which can progress to tubular necrosis (16). Cadmium poisoning can also affect other systems, including the skeletal, cardiovascular, and digestive systems, among others. The typical skeletal system disorders identified in the study include achalasia and osteoporosis (6). Cadmium-induced cardiovascular diseases are generally acute and most commonly occur following exposure to high concentrations (17). Gastrointestinal diseases may develop when inhaled cadmium irritates the mucosa of the gastrointestinal tract, causing congestion, edema, and necrosis (8). Table 3 summarizes the clinical manifestations and diagnostic methods used for patients in detail.

### Treatment

Treatment of cadmium poisoning depends on the severity of the condition. The primary goal is to reduce the body cadmium concentration, and there are several options suitable for different clinical situations. Table 4 summarizes the treatment methods included in the case reports. For mild cadmium poisoning, disengagement from exposure may reduce cadmium concentrations in the body. For instance, in the case report involving a 28-year-old female jewelry worker, the patient’s blood cadmium level decreased from 26 μg/L to 1 μg/L (near the normal range of < 5 μg/L) within 6 months after disengagement, consistent with cadmium’s blood half-life (rapid clearance period of 2–3 months). Additionally, her urinary *β*₂-microglobulin dropped from 0.16 mg/L to 0.07 mg/L, suggesting a partial reversal of renal tubular injury (18).

**Table 4:** The treatment methods and prognosis of the case.

Literature	Patient	Treatment	Prognosis
R H Townshend/1982 (15)	Patient 1	Detachment from cadmium exposure	**Short-term results:** 1. Symptom relief: fever and chest pain gradually subsided 2. Indicator (FEV₁ 80 of predicted value) **Long-term results:** Symptoms: development of pulmonary fibrosis, restrictive ventilation disorders
Richard Wittman/2002 (18)	Patient 2	Detachment from cadmium exposure	**Symptoms:** shortness of breath, chest pain and anxiety disappeared **Indicators:** **Blood cadmium**: decreased to (<5 μg/L) (normal) **Urine β₂-microglobulin:** decreased from 0.16 mg/L (abnormal) to 0.07 mg/L (normal).
Yuichi Ando/1996 (11)	Patient 3	**Initial treatment:** oxygen support, antibiotic prophylaxis against infection **Intensive treatment after deterioration:** mechanical ventilation, glucocorticoids	**Symptom relief:** respiratory failure corrected, inflammation reduced **Indicators:** recovery of lung capacity (VC), incomplete recovery of diffusion function (DLCO), decrease in urinary β₂-microglobulin
J D Blainey/1980 (34)	Patient 4	**Basic treatment:** calcium and vitamin supplementation (calcium lactate, vitamin D tablets), surgical management of fractures **Bone pain:** NSAIDs **Pulmonary embolism:** anticoagulant therapy	**Symptoms:** initial reduction of bone pain, later **death** due to multiple fractures and cardiovascular complications (cardiac arrest) **Indicators:** decreased urinary cadmium, persistent decrease in creatinine clearance, mild elevation of urea and creatinine, serum alkaline phosphatase decreases to normal after treatment
Ayan Roy/2024 (9)	Patient 5	Carbonate supplementation Active vitamin D therapy Electrolytemanagement	**Symptomatic improvement:** bone pain reduced, but non-inflammatory pain and limited mobility of the right knee still present **Indicators:** elevated blood phosphorus and potassium, decreased creatinine
Kristin Seidal/1993 (14)	Patient 6	**Antibiotic therapy:** penicillin V to benzathine penicillin **Respiratory support:** oxygen therapy **Bronchodilator: T**heophyllamine **Steroid therapy: B**etamethasone	**Died** of progressive respiratory failure Autopsy showed interstitial fibrosis
R. H. Townshend/1968 (13)	Patient 7	Not mentioned only detachment from cadmium exposure	**Symptom recovery:** chills, chest pain, respiratory distress and other symptoms disappeared **Indicators:** FVC recovered, FEV_1_/FVC remained normal, carbon monoxide diffusing capacity (DLCO) was normal.
Hector P. Blejer/1966 (16)	Patient 8	1. Antibiotics (penicillin V to benzathine penicillin) 2. Codeine for cough 3. Nebulizer and rehydration	**Died** of pulmonary edema and pulmonary hemorrhage
	Patient 9	Respiratory support (positive pressure oxygen therapy) Antibiotics (broad-spectrum antibiotics) Steroid therapy (betamethasone) Supportive measures (bronchodilators)	**Rapid symptomatic relief** **Indicators:** blood cadmium, urine cadmium back to normal indicators
D. C. Beton/1966 (23)	Patient 10	Antibiotic	Ineffective treatment, **died** after 5 days Autopsy: necrosis of lungs and kidneys
	Patient 11	Respiratory support (positive pressure oxygen therapy), Antibiotics (penicillin), Steroid therapy, Supportive measures (activity restriction)	Clinical symptoms resolved Indicators all decreased
	Patient 12		Mild anemia, mildly elevated urine protein, elevated serum protein-binding sugars
	Patient 13		Clinical symptoms resolved Indicators all decreased
	Patient 14		Clinical symptoms resolved Indicators all decreased
A. Taylor/1984 (8)	Patient 15	Chelation therapy, Steroid therapy, Positive pressure oxygen therapy	**Exacerbation of symptoms:** progressive exacerbation of pulmonary edema with cyanosis and hypoxemia. **Death** 72 h after exposure
D H Yates/1990 (5)	Patient 16	**Glucocorticoid therapy:** prednisone 40 g daily with subsequent reductions	**Short-term results:** Symptom relief: dyspnea disappeared **Long-term results:** Pulmonary function: FEV₁ 3.8 L, FVC 5.5 L Renal function: blood creatinine 9 μmol/L Creatinine clearance 152 mL/min Urinary cadmium: 1.3 nmol/mmol Cr Retinol-binding protein normal
ZHOU Yuan-shen/20220 (17)	Patient 17	**Acute life support:** Respiratory support Circulatory support, Renal support, Liver and coagulation maintenance **Specific detoxification therapy:** Chelation (CaNa₂EDTA) Traditional Chinese Medicine: Siwei Tang, tonifying qi and withdrawing yellow **Rehabilitation intervention:** Baduanjin	**Symptom relief:** 5 days later, meaning gradually awake, no obstacles to movement **Indicators:** cardiac EF recovered, blood routine normal, blood creatinine 84 μmol/L, ALT 8 U/L, BNP 297.7 pg./mL

In cases of cadmium poisoning with high cadmium concentrations, clinical management typically categorizes such cases into acute poisoning and chronic poisoning. In acute poisoning, supportive therapy and chelation therapy are commonly used to manage multi-organ dysfunction. However, chelating agents such as ethylenediaminetetraacetic acid (EDTA) while effective in reducing blood cadmium levels, also carry side effects that can cause varying degrees of damage to human organs. Given the significant adverse effects, the widespread use of such agents remains a matter of consideration. Additionally, clinical trials must demonstrate that their clinical benefits can produce favorable outcomes for all cadmium poisoning patients. Therefore, strict monitoring of the patient’s condition and a cautious risk–benefit assessment are required prior to use.

In order to mitigate the negative effects associated with chelation therapy, it is recommended to use other agents in combination during treatment to reduce its negative effects, and to investigate whether other alternative therapies can be more effective.

For chronic cadmium poisoning,traditional Chinese medicine is effective in lowering cadmium concentrations. In severe cases, Chinese medicines (e.g., Si-Yi Tang to improve shock), combined with rehabilitation exercises (Baduan Jin), may help to promote recovery (17). For patients with respiratory symptoms, oxygen therapy and pulmonary ventilation are needed to maintain normal respiratory function. Renal replacement therapy (CRRT) may be required for those with acute kidney injury or abnormal renal function. Electrolytes and acid–base disturbances should be corrected with appropriate supplementation.

Currently, the clinical treatment of cadmium poisoning faces numerous challenges. First, there are very few reported cases of cadmium poisoning. Second, the clinical symptoms exhibited by cadmium poisoning patients are scattered, so the results are only for reference and cannot be used as high-level evidence. In addition, the long-term efficacy of treatment is still unclear, and cadmium poisoning is easily confused with diseases such as lung cancer and metal fever, all of which increase the difficulty of treating cadmium poisoning.

### Outcome and follow-up

The outcome and follow-up of cadmium poisoning vary depending on the severity of the condition and treatment provided. Timely and appropriate treatment often leads to a good prognosis. For patients with acute poisoning, removal from exposure and supportive therapy can lead to a good prognosis. However, serious complications such as multiple organ failure (MODS), renal edema, and damage to the renal cortex can occur, leading to high mortality (17). In patients with chronic poisoning, renal injury is common and may progress to Fanconi syndrome during follow-up.

In some cases, the outcome is fatal, usually due to unrecognized cadmium poisoning. Cadmium poisoning can be misdiagnosed as pneumonia-related illness or metal fume fever. Table 4 provides a comprehensive summary of the prognosis of patients.

## Discussion

This review provides a comprehensive overview of cadmium poisoning. In terms of gender distribution, males were predominant among the reported cases; however, this does not mean that males are more susceptible to cadmium poisoning. In the 12 case reports, a higher proportion of males were engaged in occupations such as welding and metalworking. Therefore, it is hypothesized that cadmium poisoning is highly correlated with occupational exposure rather than gender.

Several key factors may exacerbate the symptoms of cadmium poisoning. Poor lifestyle habits, such as smoking and alcohol intake, are important aggravating factors. Smoking and alcohol consumption are known to worsen the symptoms of cadmium poisoning and significantly increase the risk of severe cases (19). A study showed that smoking increased the risk of microalbuminuria by 5.147 times (95% CI, 2.568–10.314, *p* < 0.001), and people who drink alcohol had a higher prevalence of microalbuminuria compared to non-drinkers (44.4% vs. 27.0%, *p* = 0.003). The increased incidence of proteinuria suggests a higher likelihood of nephropathy (20). In addition, low iron storage in the body can lead to increased absorption of cadmium from the intestinal tract, resulting in higher blood and urine cadmium concentrations and a greater overall body burden (21). Animal studies have also shown that alcohol intake increases urinary cadmium excretion in cadmium-exposed mice compared to non-drinking controls (22).

Diagnosis of cadmium poisoning is particularly challenging because symptoms such as shortness of breath, cough, and chest pain closely resemble those of common pneumonia and other respiratory illnesses. In the case report mentioned above, a patient with cadmium poisoning was misdiagnosed as having chemical metal fume fever, which led to a missed opportunity for optimal treatment (11). Therefore, biochemical testing is essential for identifying cadmium poisoning, as elevated levels of cadmium in blood and urine can confirm the diagnosis.

Patient-specific symptoms such as osteomalacia and elevated urinary protein require close attention. In one case report, a patient had elevated levels of FGF-23, which is associated with the development of osteomalacia. The presence of proteinuria indicated renal tubular dysfunction (9, 13). These findings reflect the pathological damage of organs linked to cadmium poisoning, i.e., the development of complications. Therefore, during follow-up and subsequent evaluations, it is also important to monitor changes in the patient’s physical condition and observe the emergence of complications and sequelae. In fatal cases, autopsy provides a definitive diagnosis of cadmium poisoning. Autopsy results indicate that death in cadmium poisoning is due to lesions in the lungs, kidneys, and other organs (23).

The treatment of cadmium poisoning involves reducing the cadmium concentrations in various organs of the body by using chelating agents. This involves using EDTA (calcium sodium ethylenediaminetetraacetic acid), which forms a complex with the cadmium ions, to promote the elimination of the ions. However, EDTA has been reported to be toxic to the kidneys. Studies have shown that EDTA increases urinary cadmium excretion, but does not affect cadmium bound to metallothionein (MT; mainly stored in the liver and kidney). However, it may lead to a sudden increase in cadmium concentration in the proximal tubule of the kidney by forming complexes with cadmium in the blood, exacerbating renal injury. EDTA has a dual effect: in acute experiments, EDTA increased urinary cadmium excretion (+35%). On the other hand, renal cortical cadmium concentration increased by 22% because the Cd-EDTA complex was actively reabsorbed by the renal tubules (via the OAT1 transporter) (10). During acute cadmium poisoning, the amount of cadmium ingested is relatively small (compared to the amount ingested in chronic kidney cadmium damage), and chelation therapy can rapidly reduce cadmium levels in the body, thereby mitigating the harm caused by heavy metals to the human body. This explains why EDTA therapy holds a priority position in the treatment of acute cadmium poisoning. However, the cadmium-EDTA complex is excreted through the kidneys via urine, and the kidneys release a small amount of cadmium. Therefore, this method requires monitoring of renal function (10). Furthermore, it has a limited effect on chronic accumulation (24). Animal studies have also found that EDTA may exacerbate tubular injury, which limits its clinical use (25).

In addition to nephrotoxicity, cadmium ion levels may also affect calcium ion balance in the body. In an animal experiment, a single intravenous injection of cadmium chloride was administered to animals at doses of 0.01, 0.015, and 0.02 millimoles per kilogram of body weight. Six hours later, serum calcium levels decreased by 2 to 4 milligrams per 100 milliliters. In surviving animals, levels remained low after 24 h. Additionally, an epidemiological study found an association between cadmium levels and cardiovascular disease. In a Korean epidemiological study, an increase in the interquartile range (IQR) of blood cadmium levels (0.91 micrograms/liter) was associated with an increased risk of IHD [OR 2.1, 95% confidence interval (CI) 1.3–3.4] (26).

In addition to the aforementioned negative clinical effects of EDTA, its efficacy remains questionable. In a large-scale randomized clinical trial (TACT2) conducted in the United States and Canada, 959 patients with a history of myocardial infarction and diabetes were enrolled to compare the effects of EDTA chelation therapy versus placebo on cardiovascular events. The results showed that the chelation group experienced a significant reduction in blood lead levels, but there was no significant difference between the chelation group and the placebo group in terms of the primary endpoint, cardiovascular mortality, and all-cause mortality. This clinical trial confirmed that EDTA has no actual benefits, further deepening clinical doubts about the feasibility of EDTA therapy.

Although EDTA therapy has a certain effect in treating heavy metal poisoning, there is considerable controversy over its clinical feasibility and potential negative effects, and whether it can be used to treat other diseases. For non-poisoning-related uses, such as coronary artery disease, this treatment method has not yet been approved by the FDA (27).

In response to the limitations of EDTA in the treatment of cadmium poisoning, some scholars have proposed the combined use of glutathione to prevent renal toxicity. In an experiment evaluating the efficacy and renal protective effects of glutathione in the chelation therapy process of chronic cadmium poisoning using (++)-ethylenediaminetetraacetic acid, it was found that the cadmium excretion in the kidneys of the patients in the experiment significantly increased. (23.4 ± 15.81 μg/g creatinine vs. 89.23 ± 58.52 μg/g creatinine, *p* < 0.01). There was no difference in the protein/creatinine and *β*(2)-microglobulin/creatinine ratio in the urine (*p* > 0.05) among the groups. Furthermore, microhematuria and proteinuria did not develop over the observation period of 6 months. These results suggest that glutathione administration with EDTA might be an effective treatment modality for patients with cadmium intoxication (35). Concurrent use of mannitol (28), thiamine (29), methionine (30). Antioxidants such as DMSA can also enhance therapeutic efficacy. In response to concerns about renal tubular damage associated with EDTA therapy, some experts have proposed sauna therapy, which involves storing cadmium in sweat and excreting it during the sauna process. Although the rate of excretion is slower than that of EDTA chelation therapy, it has a significant effect in cases of chronic cadmium poisoning with renal tubular damage (31).

Symptoms of chronic cadmium exposure include osteomalacia and renal tubular phosphorus loss, which can be alleviated with the use of calcium and vitamin D supplements (9, 13). Oxygen therapy and mechanical ventilation are commonly used in patients with lung injury or pulmonary edema.

In a study, rats were poisoned with cadmium acetate for 12 weeks and then treated with an oxygen-ozone mixture by intraperitoneal injection in the last 10 days of the experiment. An oxygen-ozone mixture had a protective effect on the liver and myocardium of cadmium-poisoned rats, as evidenced by weaker destructive changes in the endoplasmic reticulum, basal cytoplasm, and lysosomes of hepatocytes, and stabilization of contractile apparatus fibers in cardiomyocytes (32). When acute kidney injury occurs, renal replacement therapy (CRRT) is usually used. Meanwhile, the case results also found that glucocorticoid therapy helps to reduce lung inflammation, inhibit fibrosis, and ameliorate acute lung injury (33). Additionally, rehabilitation with Chinese herbal medicine is also effective in cadmium poisoning. A combination of Chinese and Western medicine has been attempted, using Si-Reversal Tang (epiphyllum, dried ginger, and licorice) to restore yang and save the body in the early stages. In later stages, astragalus and poria are used to replenish qi and benefit the gallbladder, which can ameliorate shock and hepatic injury. However, there are no controlled studies to validate this evidence. Eight-duanjin also failed to reverse organic damage (17).

The outcome of cadmium poisoning varies widely. Most patients have a better prognosis when diagnosed early, immediately removed from cadmium-exposed environments, and the cadmium levels in the body are reduced. However, death from acute cadmium poisoning as a result of organ failure is common, especially in older individuals, where increased sensitivity to cadmium toxicity contributes significantly to their death (18). Follow-up ranged from a few months to 17 years. It is worth that during the 17 years follow-up, patients subsequently developed delayed pulmonary fibrosis, disproving the previous view that denied the existence of permanent damage from acute cadmium pneumonia (15). At the same time this highlights the need for ongoing management and monitoring, especially in cases where chronic cadmium poisoning leads to respiratory failure or renal failure.

Several factors influence the outcome of treatment, including the patient’s health status, the duration of exposure to cadmium, the concentration of cadmium at the time of exposure, and the promptness and accuracy of clinical intervention. While many patients respond well to appropriate treatment, those with renal involvement often have more pronounced symptoms. Case reports indicate that acute cadmium poisoning can cause necrosis of the renal cortex and degeneration of the renal tubules (8), whereas chronic exposure results in proximal tubular dysfunction (e.g., proteinuria, glycosuria, and hypophosphatemia) and may progress to chronic kidney disease over time (18). These findings underscore the need for individualized treatment plans and comprehensive long-term follow-up to optimize patient outcomes.

Based on our results, we propose several recommendations for clinicians managing patients with cadmium poisoning. In cases where the patient has not highlighted a history of cadmium exposure, prioritize biochemical tests (urine cadmium and blood cadmium tests). These tests provide the most accurate and direct assessment of cadmium concentration in the body and help to reduce the risk of misdiagnosis, particularly when respiratory symptoms could be mistaken for pneumonia. This is because the levels of cadmium in the body are the most accurate and direct criterion for determining whether cadmium poisoning is present. However, in the face of non-specific symptoms and complications in different patients, clinicians should always be aware of the possible need to use complementary diagnostic tools, such as chest X-ray or ultrasound in patients with severe respiratory symptoms, and renal ultrasound in cases where biochemical testing reveals grossly elevated urinary cadmium levels. Early and accurate diagnosis is critical to timing treatment and preventing complications.

Long-term consequences of cadmium poisoning may include lung damage and kidney damage. Early recognition and management are essential to stop the progression of the disease. Long-term follow-up, with regular imaging evaluations, is necessary to monitor recovery and address any persistent or emerging health issues. In one case report, a 4-year follow-up chest X-ray of the reported cadmium-intoxicated patient ultimately ruled out permanent pulmonary fibrosis (13). Regular follow-up should assess the effectiveness of treatments, such as reducing cadmium concentrations and monitoring for complications. Clinicians should remain vigilant for any long-term effects or sequelae related to cadmium poisoning, particularly those affecting the kidneys and lungs.

Accurate and timely diagnosis of cadmium poisoning is crucial given the potential for rapid clinical deterioration. Cadmium poisoning occurs in several occupationally exposed populations, with certain occupational groups being at an increased risk. This underscores the importance of ongoing vigilance in these high-risk groups. Early recognition of symptoms and timely management can significantly improve patient outcomes and help prevent serious complications and sequelae.

## Limitations

A significant limitation of this systematic review is the small number of cases included, which reduces confidence in the broader applicability of the findings. In addition, there are notable gaps in data from the included studies, particularly concerning follow-up and long-term treatment outcomes. Several studies emphasized short-term responses and immediate treatment effects, providing limited information on the long-term prognosis of cadmium poisoning. In addition, there is considerable variation in reporting and treatment approaches, reflecting the lack of standardized protocols.

## Conclusion

This systematic evaluation of case series and case reports highlights the complexity and variability of cadmium poisoning, a rare condition with poor prognosis. The presence of non-specific symptoms, which are easily mistaken for other diseases, along with multiple risk factors and a range of possible complications, makes the diagnosis, treatment, and monitoring of cadmium poisoning especially challenging.

Currently, clinical and biochemical examinations remain the main definitive approaches. Depending on the symptoms, additional tests such as renal and bone density examinations may be recommended. Reducing cadmium concentration in the body is central to treatment; however, there is still no optimal method for effectively removing cadmium ions due to significant side effects of the available chelating agents.

Prognosis varies widely among patients. While many achieve good outcomes, some develop lasting complications or sequelae, underscoring the need for individualized treatment plans and rigorous follow-up care.

Future research should focus on large-scale, multicenter clinical trials to establish specific diagnostic criteria and evidence-based treatment guidelines for cadmium poisoning. This will help improve early identification, management, and long-term care for affected patients.

Cadmium poisoning represents a critical occupational and public health challenge that necessitates comprehensive preventive strategies. Policy makers and regulatory authorities should prioritize reducing cadmium use through safer alternatives, enforcing workplace safety standards such as adequate ventilation and provision of protective equipment, and implementing routine biomonitoring for high-risk occupational groups. Furthermore, strengthening legislation and regulatory oversight of cadmium-related industries, together with improving diagnostic and therapeutic capacity within medical and rehabilitation institutions, is essential to mitigate long-term health consequences and reduce disease burden.

## Data Availability

The original contributions presented in the study are included in the article/supplementary material, further inquiries can be directed to the corresponding author.

## Footnotes

^1^Available online at: https://view.officeapps.live.com/op/view.aspx?src=https%3A%2F%2Fjbi.global%2Fsites%2Fdefault%2Ffiles%2F2021-10%2FChecklist_for_Case_Reports.docx&wdOrigin=BROWSELINK
